# Comprehensive Transcriptome Analysis of Six Catfish Species from an Altitude Gradient Reveals Adaptive Evolution in Tibetan Fishes

**DOI:** 10.1534/g3.115.024448

**Published:** 2015-11-10

**Authors:** Xiuhui Ma, Wei Dai, Jingliang Kang, Liandong Yang, Shunping He

**Affiliations:** * The Key Laboratory of Aquatic Biodiversity and Conservation of Chinese Academy of Sciences, Institute of Hydrobiology, Chinese Academy of Sciences, Wuhan, Hubei, 430072, China; † School of Life Science, Southwest University, Beibei, Chongqing, 400715, China; ‡ Institute of Hydrobiology, University of Chinese Academy of Sciences, Beijing, 10001, China

**Keywords:** Tibetan Plateau, adaption, gradient altitudes, comprehensive transcriptome, glyptosternoid fishes, accelerated genic evolution

## Abstract

Glyptosternoid fishes (Siluriformes), one of the three broad fish lineages (the two other are schizothoracines and *Triplophysa*), have a limited distribution in the rivers in the Tibetan Plateau and peripheral regions. To investigate the genetic mechanisms underlying adaptation to the Tibetan Plateau in several fish species from gradient altitudes, a total of 20,659,183–37,166,756 sequence reads from six species of catfish were generated by Illumina sequencing, resulting in six assemblies. Analysis of the 1,656 orthologs among the six assembled catfish unigene sets provided consistent evidence for genome-wide accelerated evolution in the three glyptosternoid lineages living at high altitudes. A large number of genes refer to functional categories related to hypoxia and energy metabolism exhibited rapid evolution in the glyptosternoid lineages relative to yellowhead catfish living in plains areas. Genes showing signatures of rapid evolution and positive selection in the glyptosternoid lineages were also enriched in functions associated with energy metabolism and hypoxia. Our analyses provide novel insights into highland adaptation in fishes and can serve as a foundation for future studies aiming to identify candidate genes underlying the genetic basis of adaptation in Tibetan fishes.

The mechanisms underlying organismal adaptation to high-altitude hypoxia have recently become of great interest (Gou *et al.* 2014). The Tibetan Plateau (the ‘‘Roof of the World’’) is the highest plateau on Earth, with an average elevation of more than 4000 m. The plateau covers more than 2,500,000 km^2^ of plateaus and mountains in central Asia and is surrounded by towering mountain ranges. Despite its inhospitable environment, various adaptive responses that may be responsible for highland adaptation have been identified in several species, including Tibetans (Beall *et al.* 2010; Bigham *et al.* 2010; Simonson *et al.* 2010; Yi *et al.* 2010; Peng *et al.* 2011), yak (Qiu *et al.* 2012), Tibetan antelope (Ge *et al.* 2013), Tibetan wild boar (M. Li *et al.* 2013), ground tit (Qu *et al.* 2013), Tibetan mastiff (Gou *et al.* 2014; Li *et al.* 2014), and a schizothoracine fish (Yang *et al.* 2015). Among these adaptive processes, genes exhibiting signs of positive selection and expansion were significantly enriched in hypoxia and energy metabolism pathways. However, almost all previous genome-wide studies were performed on endothermic terrestrial vertebrates. Little is known about the genomic basis of the adaptation of fishes to highland regions, with the exception of the schizothoracine fishes, which only include one species from a high-altitude environment (Yang *et al.* 2015). Therefore, investigating the mechanism of adaptation to the conditions of the Tibetan Plateau in several fishes from gradient altitudes may provide novel insights.

Glyptosternoid fishes (Sisoridae, Siluriformes), one of the three broad fish lineages (the two other are schizothoracines and *Triplophysa*), exhibit a limited distribution in the rivers of the Tibetan Plateau and peripheral regions. These fishes provide an excellent model system in which to study how fishes adapt to high-altitude habitats. They form a lineage that is distributed in the rivers around the Tibetan Plateau and eastern Himalayas [*e.g.*, the Yaluzangbujiang (Brahmaputra River), Irrawaddy, Nujiang (Upper Salween), Lancangjiang (Upper Mekong River), Jinshajiang (Upper Yangtze), Yuanjiang (Red River), Nanpanjiang (Upper Pearl River), and the Brahmaputra basin] (Guo *et al.* 2005) and the downstream portions of these rivers in India, Burma, Thailand, Laos, Bangladesh, and Vietnam. The glyptosternoid fishes are extremely well adapted to the rapidly flowing water environment and possess a series of adaptive structures, including a depressed body and head, horizontally inserted pectoral and ventral fins, and an adhesive apparatus on their paired fins, which are suited for this environment (Guo *et al.* 2005; Peng *et al.* 2006). The fishes (*Glyptosternon maculatum*, *Pareuchiloglanis sinensis*, and *Pareuchiloglanis macrotrema*) studied here are freshwater catfish that belong to the Glyptosternoid fishes that inhabit the cold waters of the highlands. *Glyptosternon maculatum*, the base of the Glyptosternoid fishes, inhabits shallow, rocky rivers with a moderate current, where it feeds on invertebrates. It occurs only in the middle-reach of the Yaluzangbu River in Tibet, and in the Brahmaputra River in India. *Pareuchiloglanis sinensis* and *Pareuchiloglanis macrotrema* are found only in the Jinshajiang (upper Yangtze) and Yuanjiang (Red River), respectively.

The objective of this study was to undertake a comparative transcriptome-wide search for genes that might be involved in adaptation to high-elevation environments and to identify their associated functions in six catfish from gradient altitudes. We sampled liver tissues of *G. maculatum*, *P. sinensis*, *P. macrotrema*, Amur catfish (*Silurus asotus*), yellowhead catfish (*Pelteobagrus fulvidraco*), and *Synodontis nigriventris* and sequenced their transcriptomes using an Illumina sequencing platform. We identified genes showing strong signs of positive selection by comparing their transcriptomes. The functional and phenotypic outcomes of these candidate genes were inferred by annotation based on genomic resources of a variety of model vertebrate species. We report a list of candidate genes that are highly likely to be involved in high-elevation adaptation processes.

## Materials and Methods

### Ethics statement

The methods involving animals in this study were carried out in accordance with the Laboratory Animal Management Principles of China. All experimental protocols were approved by the Ethics Committee of the Institute of Hydrobiology, Chinese Academy of Sciences.

### Fish sampling

**Figure 1:**
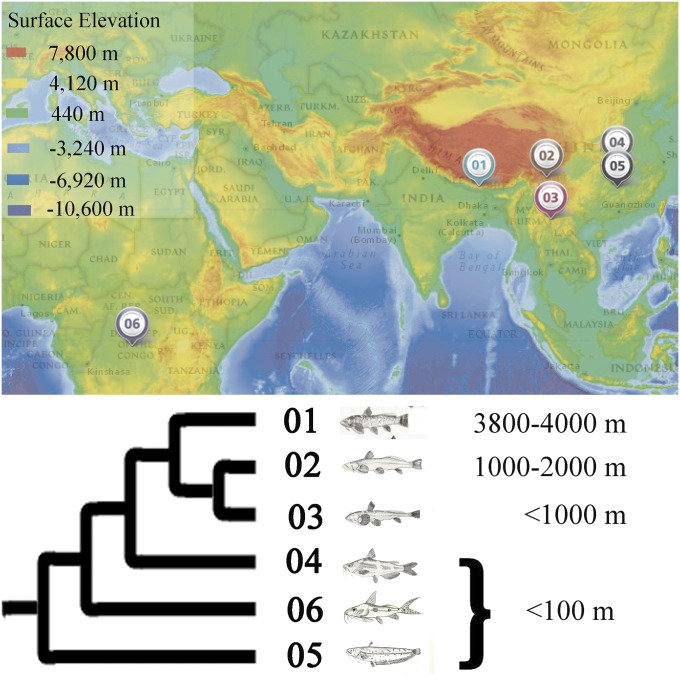
Altitude distribution of six catfish species. Map from National Geographic’s MapMaker Interactive on April 13, 2015 (http://education.nationalgeographic.com/education/mapping/interactive-map/?ar_a=1); the lower of Surface Elevation was added. Details on the species are provided in Table S1. The tree inferred using 1656 orthologous gene with the Bayesian inference (BI) and maximum likelihood (ML) method is shown below the map.

We sampled three glyptosternoid fishes, including *G. maculatum* from the Yaruzampbo River in Tibet (3800–4000 m), *P. sinensis* from Daduhe River in Sichuan (1000–2000 m), and *P. macrotrema* from the Yuan River in Yunnan (<1000 m), as well as three species from low altitude (details in Figure 1, and Supporting Information, Table S1). Samples were taken back to the laboratory, where each species of fish were stocked into one aquarium. All aquaria were maintained under a natural photoperiod, water temperature fluctuated from 10° to 15°, and pH ranged from 6.5 to 7.5. Dissolved oxygen concentration remained higher than 5 mg/L, and ammonia-nitrogen was lower than 0.01 mg/L. The acclimation study was conducted for 48 hr and all fish were starved. Then three individuals of each species were chosen and anesthetized with MS-222 (100 mg/L, Sigma Chemical Company, St Louis, MO), and liver tissues immediately collected. The tissues were flash frozen in liquid nitrogen and placed in a −80° freezer prior to processing for total RNA isolation. For each species, liver tissues from three individuals were mixed.

### RNA isolation and sequencing

We sequenced the liver tissue transcriptomes to access a large number of diverse transcripts because the liver is a highly complex organ with a complex transcriptome (Shackel *et al.* 2002, 2006). Total RNA was isolated using TRIzol reagent (Life Technologies Corp., Carlsbad, CA) according to the manufacturer’s protocols, and cleaned up using the RNeasy mini kit (Qiagen, Valencia, CA). RNA samples were quantified with the 2100 Bioanalyzer (Agilent Technologies). mRNA was enriched using beads with oligo (dT) and fragmented using fragmentation buffer. The first cDNA chain was synthesized using random hexamers, and the second chain was synthesized with buffer, dNTPs, RNase H, and DNA polymerase I. After the cDNA was purified using Agencourt AMPure XP (Beckman Coulter Inc., Atlanta, GA), the samples were blunt-end repaired and ligated with poly(A) and adapter sequences. Then, sizes were selected using Agencourt AMPure XP (Beckman Coulter Inc., Atlanta, GA). Each of the libraries was amplified by PCR. The libraries were quantified by RT-PCR, and transcriptome sequencing was performed on an Illumina HiSequation 2500 platform. Short sequence reads of paired end (PE) 100 bp were generated. All of the data were deposited into the NCBI Sequence Read Archive database (SRA run accession numbers are provided in Table S1).

### *De novo* assembly and gene functional annotation

These reads were then filtered, and defective reads were removed for each species. High-quality sequencing reads and appropriate assembly methods are essential for obtaining a reliable *de novo* assembly, which serves as the foundation of all subsequent analyses (Robertson *et al.* 2010; Surget-Groba and Montoya-Burgos 2010). First, quality control checks were conducted on the raw sequence data using FastQC (http://www.bioinformatics.bbsrc.ac.uk/projects/fastqc). The adapter sequences and sites with lower qualities (Phred score < 20) reads were trimmed using the Cutadapt (Martin 2011). All subsequent analyses were based on these cleaned reads. *De novo* sequence assembly was performed using Trinity (Grabherr *et al.* 2011) software designed for the assembly of short read sequences with default parameters. Only contigs with lengths greater than 200 bp were used for further analysis. To lower the redundancy in the dataset, low-coverage artifacts or redundancies were removed using the CD-HIT-EST program (Li and Godzik 2006) with an identity threshold of 95%.

To annotate the assembled unigenes, we downloaded the zebrafish (*Danio rerio*) protein dataset from the Ensembl database (http://useast.ensembl.org, release 78), and then used BLASTx searches to map the unigenes to the proteins with an E-value cutoff of 1e–10. Putative functions for the assembled unigenes were assigned by the Blast2GO suite (Gotz *et al.* 2008) using BLASTx against the nonredundant (nr) database with a conservative E-value cutoff of 1e–5.

### Orthology determination

We used HaMStR (version v.13.2.3) to infer orthology (Ebersberger *et al.* 2009), which in turn used usearch (http://drive5.com/usearch), GeneWise (Birney *et al.* 2004), and HMMER (Eddy 2011) to search the combined assembly data for protein sequences matching a set of “customl” orthologs. The “customl” orthologs in our case consisted of a database of eight genomes representing Ostariophysi fishes: Cypriniformes, *D. rerio* (DANIO); Tetraodontiformes, *Takifugu rubripes* (TFUGU); Beloniformes, *Oryzias latipes* (MEDAK); Perciformes, *Oreochromis niloticus* (ONILE); Cyprinodontiformes, *Xiphophorus maculatus* (SWORD); Gasterosteiformes, *Gasterosteus aculeatus* (STIKLE); Tetraodontiformes, *Tetraodon nigroviridis* (TETRA); and Gadiformes, *Gadus morhua* (ANCOD). If the unigenes were assigned to different “custom” orthology sequences, the longest unigene was chosen.

One-to-one orthologs between six catfish and zebrafish were determined using the reciprocal BLAST best-hit method with an E-value cutoff of 1 × 10^−10^. Then, putative single copy orthologs among six catfish were obtained. The longest transcript was chosen for genes with multiple transcripts. Each orthologous gene set was aligned using GeneWise (Birney *et al.* 2004), and trimmed using Gblocks (Castresana 2000) with the parameter “–t = c.” We further deleted all gaps and “N” from the alignments to reduce the effect of ambiguous bases on the inference of positive selection. After the deletion process, trimmed alignments shorter than 150 bp (50 codons) were discarded from subsequent analyses.

### Substitution rate estimation and selection analyses

To estimate the lineage-specific evolutionary rates for each branch of the six species, the Codeml program in the PAML package (Yang 2007) was run with the free-ratio model (model = 1) for each ortholog, a concatenation of all alignments of the orthologs, and 1000 concatenated alignments constructed from 10 randomly chosen orthologs. Parameters including dN, dS, Ka/Ks, N*dN, and S*dS values were obtained for each branch, and genes were discarded if N*dN or S*dS < 1 or dS > 1 according to the method described in a previous study (Goodman *et al.* 2009). We used the branch model to identify fast-evolving genes, with the null model assuming that all branches have been evolving at the same rate and the alternative model allowing foreground branches to evolve under a different rate. The likelihood ratio test (LTR) with df = 1 was used to discriminate between alternative models for each ortholog in the gene set. Multiple testing was corrected by applying the false discovery rate method (FDR) implemented in R (Storey and Tibshirani 2003). We considered the genes to be evolving with a significantly faster rate in the foreground branch if the FDR-adjusted *P* value was less than 0.05, and a higher ω value was detected in the foreground branch than the background branches. To detect positive selection on a few codons along specific lineages, we used the optimized branch-site model (Zhang *et al.* 2005) following the author’s recommendations. A likelihood ratio test was used to compare a model that allowed sites to be under positive selection in the foreground branch with the null model, in which sites could evolve neutrally and under purifying selection. The p-values were computed based on the chi-square statistic adjusted by the FDR method; genes with adjusted *P* values < 0.05 were treated as candidates for positive selection. Gene ontology (GO) functional enrichment analyses for both fast-evolving genes and positively selected genes were performed by DAVID (Dennis *et al.* 2003).

### Data availability

The sequencing reads were deposited in the short read archive (SRA) of GenBank (accession numbers listed in Table S1).

## Results

### Illumina sequencing, *de novo* assembly, and sequence validation

A total of 20,659,183–37,166,756 sequence reads were generated from the six catfish by Illumina sequencing (for detailed information, see Table S2). First, we filtered these reads and removed the defective reads for each species. High-quality sequence reads and the use of appropriate assembly methods are essential to obtaining reliable *de novo* assembly, which is the foundation for all other analyses (Surget-Groba and Montoya-Burgos 2010; Robertson *et al.* 2010; Garg *et al.* 2011). In total, six raw assemblies were obtained for the six catfish and further merged by integrating sequence overlaps and eliminating redundancies for each species. Detailed information on the total length of the final set of assemblies and unigene numbers is summarized in Table S3; the length distribution of all unigenes is shown in Figure S1.

We used gene coverage and transcript sequence quality to assess how well the assembled sequences represented the actual transcriptome population. The transcriptome gene coverage was judged by comparing it with the sequence information available for *G. maculatum*. All 13 mitochondrial protein-coding genes in the NCBI database were present in full length in our assembled transcripts. Next, we compared our assembled scaffolds with the zebrafish transcriptome (ENSEMBL Zv9) and found that 36,832 out of 48,435 (76.04%) zebrafish transcripts had matches in the assembled contigs. At the same time, 14,828 reciprocal best-hit BLAST matches with the zebrafish transcriptome were identified using an E-value of 1e–5. Transcriptome quality was assessed by comparing the mitochondrial protein-coding genes found in the assembled sequences to the mitochondrion sequence in GenBank (NC_021597). A total of 11,409 nucleotide identities were observed out of the 11,398 bp (99.9%) total nucleotide length of the contig to the coding mitochondrial sequences in the BLAST matches, suggesting very good transcriptome sequence quality. The observed 0.1% sequence difference might be due to high intraspecific genetic variability. The same assembly quality was found for the other five catfish species.

### Gene annotation

We used several complementary approaches to annotate the assembled unigenes. First, a BLASTx search against zebrafish proteins returned an average of 25.5–49.3% catfish unigenes with significant hits to zebrafish genes (Table S4 and Figure S2). The average percentages of unigenes with BLAST hits are similar to previous *de novo* transcriptome studies of nonmodel organisms (Guo *et al.* 2013; Yang *et al.* 2015). Unigenes without significant hits may consist of orphan genes, noncoding RNAs, untranslated transcripts, or misassembled transcripts. Second, we used Blast2GO with the GO annotation database to assign their putative functions (Figure S3). Finally, clusters of orthologous groups of proteins (COGs) and the nonredundant (NR) databases were used to further annotate these unigenes and to produce good results for putative proteins (Table S5).

### Identification of putative orthologs

To better understand the evolutionary dynamics of glyptosternoid fish in response to the highland environment, putative orthologs among six catfish were determined using two methods. First, the Hmmer search was used to identify putative orthologs among the six species (Bazinet *et al.* 2013). A total of 708 putative orthologs were identified by comparing all six transcript sets; a total of 170 was retained after alignment and trimming for quality control (see *Materials and Methods*).

To the reciprocal BLAST best-hit method, a total of 1656 orthologs ranging from 150 to 7155 bp were retained after alignment and trimming for quality control; these orthologs were used in the subsequent evolutionary analysis. Despite the fact that the lengths of the orthologs were shorter after trimming, the shapes of their length distributions were generally similar (Figure S4).

### Accelerated evolution in the glyptosternoid lineage

**Figure 2:**
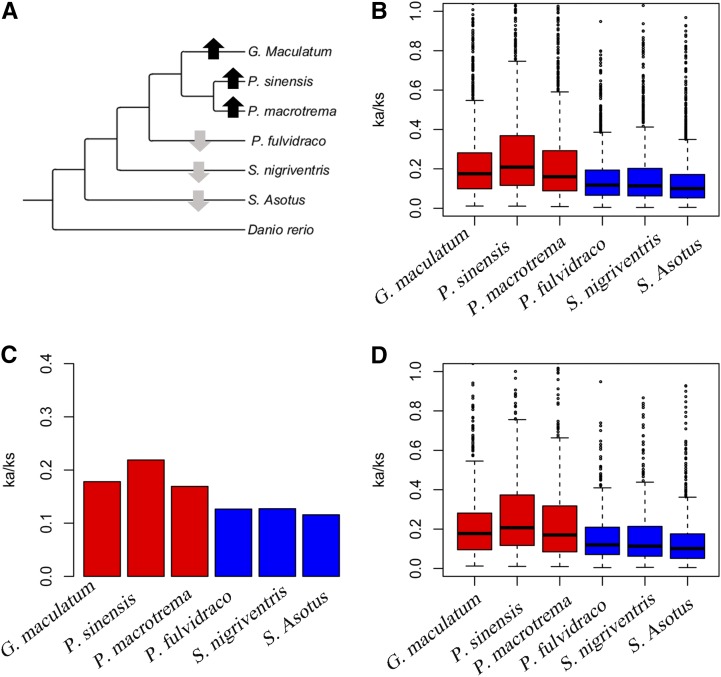
Phylogenetic tree used in this study (A), and branch-specific Ka/Ks ratios obtained from different datasets (B, C, D). Gray and black arrows in (A) indicate decreased or increased terminal Ka/Ks ratios, respectively, compared with the ancestral branch. The Ka/Ks ratios for terminal branches were estimated from each ortholog (B), all concatenated orthologs (C), and 1000 concatenated alignments constructed from 10 randomly chosen orthologs (D).

**Figure 3:**
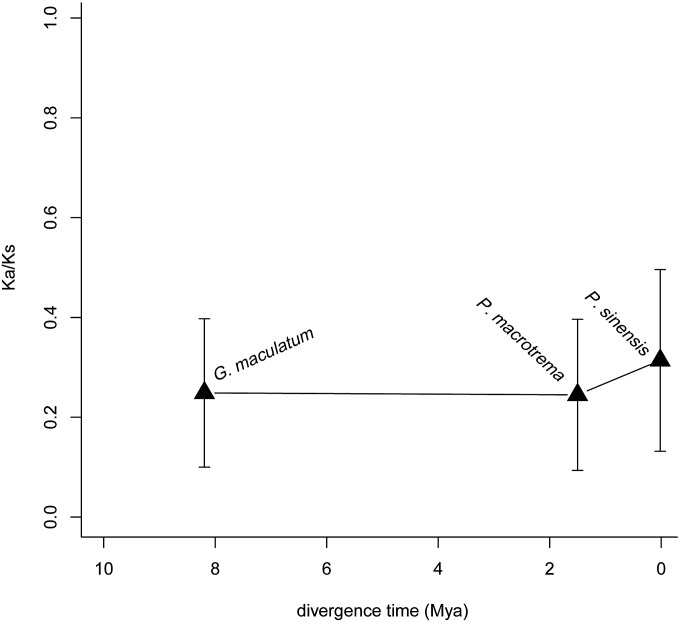
Ka/Ks ratios of the three living glyptosternoid lineages.

First, to compare the overall difference in selective constraints in the different branches at the gene level, each orthologous gene was evaluated for substitution rates (*i.e.*, Ka, Ks, and Ka/Ks) using the species tree (inferred using the BI and ML method) (Figure 2A). The free-ratio model (M1 model) in PAML was used, which allows for an independent Ka/Ks ratio for each branch. Indeed, by examining the Ka/Ks ratios for 1656 orthologous genes in the glyptosternoid lineage (Figure 2B), we found that 480 genes had higher Ka/Ks values in all three glyptosternoid fish lineages. Then, we calculated the Ka/Ks ratio for each branch in a concatenated alignment of all 1656 orthologs and 1000 concatenated alignments constructed from 10 randomly chosen orthologs and found that both datasets also exhibited a significantly higher Ka/Ks ratio for all of the living glyptosternoid lineage fish branches compared to the other catfish branches (*P* < 2.2 × 10^−16^, Figure 2, C and D). The Ka/Ks of *P. sinensis* was highest within the glyptosternoid lineage (0.3139, Figure 3). Therefore, comparison of the Ka/Ks ratios indicated accelerated evolution in glyptosternoid fish after their split from the yellowhead catfish.

**Figure 4:**
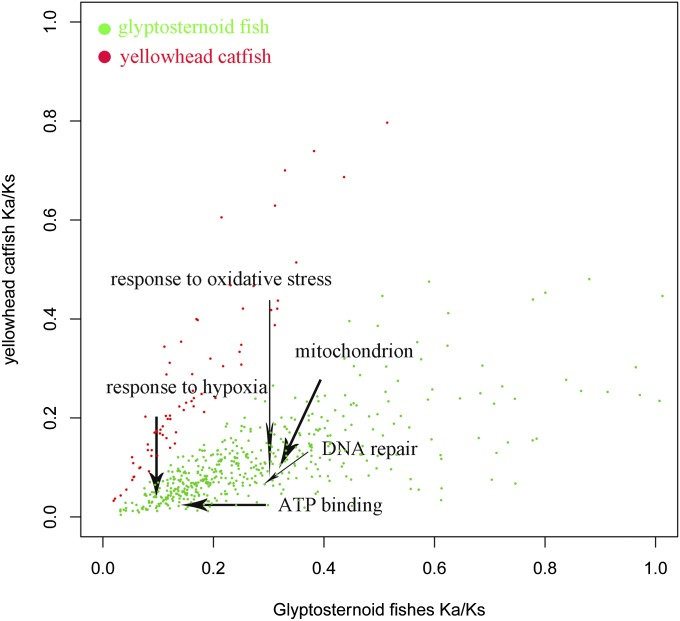
Scatter plot of the mean Ka/Ks ratios for each gene ontology (GO) category in glyptosternoid and yellowhead catfish. GO categories with significantly higher mean Ka/Ks ratios in glyptosternoid (green) and yellowhead catfish (red) are highlighted.

A number of GO categories were involved in the 480 genes in the glyptosternoid lineage that underwent rapid evolution compared to the yellowhead catfish. For example, genes associated with energy metabolism, hypoxia response, and DNA repair showed significantly accelerated evolution in glyptosternoid fish compared to the yellowhead catfish. Categories that underwent rapid evolution compared to the zebrafish included “response to hypoxia,” “response to oxidative stress,” “oxidoreductase activity,” “mitochondrion,” “ATP binding,” “GTPase activity,” and “DNA repair” (Figure 4 and Table S6).

### Fast-evolving and positively selected genes

To detect genes that might have evolved due to lineage-specific adaptation, two types of gene sets were compiled: 1) fast-evolving genes (FEGs) that exhibited a significantly higher Ka/Ks ratio in specific lineages compared with other lineages, and 2) positively selected genes (PSGs) that were influenced by positive selection only on a few codons along a particular lineage (see *Materials and Methods*). In total, we identified 121–178 FEGs in living glyptosternoid fish, 63 FEGs in yellowhead catfish and 58–244 PSGs in living glyptosternoid fish, and 48 PSGs in yellowhead catfish (Table S7 and Table S8). This finding suggests that the living glyptosternoid fish lineages have higher numbers of FEGs and PEGs compared to yellowhead catfish. Functional enrichment analysis showed that the FEGs identified in each glyptosternoid lineage were significantly enriched for genes involved in energy metabolism and oxidation-related functions, including “ATP binding,” “mitochondrial part,” “GTPase regulator activity,” “ATPase activity,” and “Acyl-CoA oxidase/dehydrogenase,” whereas FEGs detected in yellowhead catfish were generally enriched in functions involved in structure components (Table S9).

To identify genes that might directly contribute to the adaptation to high altitude, we used the 244 PSGs in *G. maculatum* as the candidate genes, then combined two approaches to annotate all of the candidate genes according to their functional roles. First, we compared our candidate genes (PSGs) to an *a priori* list proposed by Zhang *et al.* (2014), which included 1351 putative hypoxia-related genes. Second, we used the functional annotated information for each PSG to identify the genes associated with the hypoxia response reported in previous experimental studies. In total, we identified 13 candidate PSGs in glyptosternoids that may be involved in the hypoxia response: *Slc2a8*, *Igfbp7*, *C2*, *Cp*, *Ndc1*, *Hspa5*, *Ttr*, *Gapdh*, *Prmt5*, *Srebf1*, *Perp*, *Map3k14*, and *Fam162a* (Table 1).

**Table 1:** Positively selected genes involved in the hypoxia response in *Glyptosternon maculatum*

Gene ID	Gene Name	Description
ENSDARG00000020344	*Slc2a8*	Solute carrier family 2 (facilitated glucose transporter), member 8
ENSDARG00000017389	*Igfbp7*	Insulin-like growth factor binding protein 7
ENSDARG00000019772	*C2*	Complement component 2
ENSDARG00000010312	*Cp*	Ceruloplasmin (ferroxidase)
ENSDARG00000021120	*Ndc1*	NDC1 transmembrane nucleoporin
ENSDARG00000004665	*Hspa5*	Heat shock 70 kDa protein 5 (glucose-regulated protein, 78 kDa)
ENSDARG00000037191	*Ttr*	Transthyretin
ENSDARG00000043457	*Gapdh*	Glyceraldehyde-3-phosphate dehydrogenase
ENSDARG00000079605	*Prmt5*	Protein arginine methyltransferase 5
ENSDARG00000067607	*Srebf1*	Sterol regulatory element binding transcription factor 1
ENSDARG00000063572	*Perp*	PERP, TP53 apoptosis effector
ENSDARG00000063344	*Fam162a*	Family with sequence similarity 162, member A
ENSDARG00000074060	*Map3k14*	Mitogen-activated protein kinase kinase kinase 14

## Discussion

The glyptosternoid fishes distributed in the rivers of the Tibetan Plateau and peripheral regions are extremely well adapted to high altitude. These fishes are distributed across wide altitudes that range from several hundred to more than 4000 m. Thus, these species serve as an ideal model system in which to investigate genetic adaption to high altitude. However, no genome sequencing data are available, even at the order level (Siluriformes). Transcriptome sequencing represents an effective and accessible approach to initiating comparative genomic analyses on nonmodel organisms when genome sequencing data are not available because transcriptomes contain a large number of protein-coding genes that are most likely enriched for targets of natural selection. Over the past few years, comparative genomics has been widely employed as a tool to understand the genetic basis of many fundamental evolutionary questions, including adaptation (Jones *et al.* 2012; Axelsson *et al.* 2013; Zhao *et al.* 2013; Koch 2014; Zhang *et al.* 2014), speciation (Harr and Price 2012; Soria-Carrasco *et al.* 2014; Van Leuven *et al.* 2014) and genetic variation (Guo *et al.* 2012; Aflitos *et al.* 2014; Joseph *et al.* 2015). Here, we generated and annotated the first comprehensive transcriptome resource for three glyptosternoid fishes that are endemic to the Tibetan Plateau, and show many unique traits that have enabled their adaptation to highland environments compared to three other catfish species from the plains using RNA-seq technology. We generated 1656 pairwise orthologous genes between zebrafish, which served as an important basis for comparative genomic studies of adaptation in fishes. Therefore, the transcriptome resources produced by our study are useful for understanding the genetic makeup of fishes at high altitudes, and provide a foundation for further studies to identify candidate genes underlying the adaptation of fishes to the Tibetan Plateau. The glyptosternoid fishes (Siluriformes) represent one of the three broad fish lineages (including the schizothoracines and *Triplophysa*) commonly found on the Tibetan Plateau. Previous research focused on the high-altitude adaptation of the schizothoracine fish (Cyprinidae) based on the mitochondrial genome (Y. Li *et al.* 2013) and comprehensive transcriptome (Yang *et al.* 2015) of only one species. Our results were similar to the results of the schizothoracine fish study (Yang *et al.* 2015). It suggested that accelerated evolution occurred in glyptosternoid fishes living in high-altitude environments after their split from yellowhead catfish. The evolutionary rate of *P. sinensis* was highest within the glyptosternoid fish lineage, followed by *G. maculatum* and *P. macrotrema*. *G. maculatum*, the basal group of glyptosternoid fishes, is distributed widely in Brahmaputra drainages. This species was found to originate in the Late Miocene age (c. 8.2 Ma), whereas *P. macrotrema* separated from *P. longicauda* 1.5 MYA ago. *P. sinensis* is very young; this lineage formed during the Late Pleistocene (ca. 0.018 Ma) and is distributed in the Upper Yangtze River. This divergence time was inferred from 12 mitochondrial protein coding genes from 22 glyptosternoid fishes (unpublished data). Orogenic development in northern Tibet has affected the fauna, including fish and pikas (Yu *et al.* 2000; Ruber *et al.* 2004). With the uplift of the Tibetan Plateau, Chinese glyptosternoid fish speciation exploded. At the same time, *P. sinensis* may have experienced a more accelerated evolution facilitated by the Upper Yangtze River environment.

The most extreme challenge for species living in high-altitude environments is the low oxygen supply (Beall 2007). To identify the potential genes directly involved in hypoxia, we focused on the function of positively selected genes in the glyptosternoid fish lineage. We identified several interesting candidate genes that may be involved in the response to hypoxia. Among them, 13 genes were found to most likely be involved in the adaptive process to high-elevation environments, particularly genes associated with the response to hypoxia and oxygen binding. For example, solute carrier family 2 (facilitated glucose transporter) member 8 (SCL1A8) belongs to the solute carrier 2A family, which includes intracellular glucose transporters regulated by hypoxia-inducible factor-1α (HIF-1α) (Saxena *et al.* 2007; Shao *et al.* 2014). Insulin-like growth factor-binding protein-7, encoded by the *Igfbp7* gene, is involved in the regulation of the availability of insulin-like growth factors (IGFs) in tissues and modulating IGF binding to its receptors, and may suppress the stimulatory effect of vascular endothelial growth factor (VEGFA) (Tamura *et al.* 2014). Ceruloplasmin (Cp), a copper protein with a potent ferroxidase activity that converts Fe^2+^ to Fe^3+^ in the presence of molecular oxygen, is a ferroxidase that is important in the regulation of both systemic and intracellular iron levels. Cp-stimulated iron release was absolutely dependent on the presence of apotransferrin and hypoxia (Sarkar *et al.* 2003). Cp has a critical role in iron metabolism in the brain and retina, and alters intracellular iron-regulated proteins and pathways, including ferritin, transferrin receptor, glutamate and hypoxia-inducible factor-1a, through the Cp-induced nuclear translocation of the hypoxia-inducible factor-1 (HIF-1) subunit HIF-1a (Harned *et al.* 2012). NDC1, a crucial membrane-integral nucleoporin of metazoan nuclear pore complexes, is required for nuclear pore complex assembly (Mansfeld *et al.* 2006); moreover, cytoplasmic–nuclear transport of HIF-1α occurs through the nuclear pore (Nagara *et al.* 2013). Transthyretin (TTR), the thyroid hormone-binding protein, is critical for adaptation to hypoxia; the expression of this protein is upregulated in low-oxygen environments (Patel *et al.* 2012). Glyceraldehyde-3-phosphate dehydrogenase (GAPDH) is a multifunctional enzyme that is overexpressed in many tumors and is induced by hypoxia in normal and malignant cells. The degree to which hypoxia transcriptionally activates GAPDH is cell-type specific (Lu *et al.* 2002). The GAPDH promoter region contains a hypoxia responsive element (HRE) consisting of a HIF-1 consensus binding site plus adjacent sequence (Graven *et al.* 1999). Protein arginine methyltransferase 5 (PRMT5) is a novel regulator of HIF-1- and HIF-2-mediated responses. Hypoxia-inducible factors (HIF-1 and HIF-2) are essential mediators for the adaptive transcriptional response of cells and tissues to low-oxygen conditions. PRMT1 is a repressor of both HIF-1 and HIF-2. The cellular depletion of PRMT1 by small interfering RNA targeting led to increased HIF transcriptional activity. This activation was the result of enhanced HIF-α subunit transcription that allowed for increased HIF-α subunit availability. Sterol regulatory element binding transcription factor 1 (SREBF1) encodes a transcription factor that binds to the sterol regulatory element-1 (SRE1), which is a decamer flanking the low-density lipoprotein receptor gene and other genes involved in sterol biosynthesis. Sterol regulatory element binding proteins (SREBPs) are hypoxic transcription factors required for adaptation to low-oxygen environments (Todd *et al.* 2006; Bien and Espenshade 2010). The candidate genes are potentially involved in hypoxia pathways, but none are shared with previously reported genes in other fishes (Yang *et al.* 2015). This observation suggests that catfish at high altitudes may have employed a different genic toolkit to adapt to the extreme environment of the Tibetan Plateau. Moreover, several experimental studies revealed that amino acid variants in the alpha- and/or beta-globin genes can undoubtedly change Hb-O_2_ affinity in high-altitude species, including deer mice (Storz *et al.* 2009, 2010; Natarajan *et al.* 2013) and Andean hummingbirds (Projecto-Garcia *et al.* 2013). Although the genetic basis of hypoxia tolerance has yet to be fully elucidated in some vertebrate species, evidence from a number of birds, mammals, and amphibians suggests that modifications of hemoglobin (Hb) function may often play a key role in mediating an adaptive response to high altitude hypoxia (Storz 2007; Storz and Moriyama 2008). However, evidence about the mutations to adaptation to the Tibetan Plateau in Tibetan fishes was unclear. Therefore, the genes that display signatures of positive selection will serve as a baseline for further investigations that aim to understand high-elevation adaptation at both the molecular and phenotypic levels. A deep understanding of the adaptation to the Tibetan Plateau in Tibetan fishes can only be achieved by experimental and functional genomics in future. It also needs to be further confirmed by population genomics studies in the future.
